# A SNP Mutation in Homeodomain-DDT (HD-DDT) Transcription Factor Results in *Multiple Trichomes* (*mt*) in Cucumber (*Cucumis sativus* L.)

**DOI:** 10.3390/genes12101478

**Published:** 2021-09-23

**Authors:** Zhige Yang, Mengfei Song, Feng Cheng, Mengru Zhang, Marzieh Davoudi, Jinfeng Chen, Qunfeng Lou

**Affiliations:** State Key Laboratory of Crop Genetics and Germplasm Enhancement, College of Horticulture, Nanjing Agricultural University, Nanjing 210095, China; 2018104066@njau.edu.cn (Z.Y.); 2017204016@njau.edu.cn (M.S.); 2018204029@njau.edu.cn (F.C.); 2017104068@njau.edu.cn (M.Z.); 2018204052@njau.edu.cn (M.D.); jfchen@njau.edu.cn (J.C.)

**Keywords:** *Cucumis sativus* L., *multiple trichomes* mutant, genetic mapping, HD-DDT transcription factor, transcriptome

## Abstract

Trichome is a natural physical barrier protecting plants against environmental stresses, natural infestations, ultraviolet rays and pathogenicity. Trichome also helps plants in maintaining appropriate water content by reducing transpiration rate. The molecular mechanism regulating unicellular trichome development in Arabidopsis has been extensively elucidated, but the molecular mechanism regulating multicellular trichome development remains unclear. In this study, we identified a *multiple trichomes* (*mt*) mutant from a cucumber EMS (Ethylmethylsulfone) mutagenesis population. Genetic analysis indicated that an incomplete dominant gene controls the *mt* trait. Using a combination of map-based cloning and BSA-seq (Bulked Segregant Analysis -Sequencing), we identified the candidate gene, *CsaV3_6G050410*, responsible for the *mt* mutation. Sequence alignment revealed one base substitution in gene *CsaV3_6G050410*, resulting in an amino acid substitution. The deduced amino acid sequence of *CsaV3_6G050410* encodes a HD-DDT (homeodomain-DDT) transcriptional regulatory protein containing a conserved homeobox domain and a DDT domain. Gene expression analysis revealed that the expression level of *CsaV3_6G050410* in the *mt* mutant was similar to that in the WT (wild type). Transcriptome analysis indicated that the *mt* gene may regulate the development of the epidermis by influencing plant hormone signaling pathways or participating in several transcription factor pathways. The results of this study are fundamental for a better understanding of the function of the HD-DDT transcription factor in the trichome development of cucumber.

## 1. Introduction

Trichome is a specialized structure, developed from plant epidermal cells, mainly distributed on the surface of aerial organs, including leaves, stems and flower organs [1]. Trichome is mainly composed of single or multiple cells, and its size and density vary distinctively with its growth stages and locations [2,3]. Trichome plays a vital role in the plant’s defense against environmental stresses, diseases and insect pests, nutrient absorption, water loss via transpiration, ultraviolet radiation and seed protection [4,5,6]. Some plants have a glandular trichome, which can synthesize, store and secrete various metabolites, including organic acids, polysaccharides, proteins, polyphenols, alkaloids and terpenoids [7,8]. In addition, several plant trichomes have important agronomic traits, such as cotton fiber [9], and the glandular trichome of *Artemisia annua* [10].

At the genetic level, the development of plant trichome is strictly controlled by complex regulatory mechanisms. The mechanisms of unicellular trichome development in Arabidopsis have been extensively clarified. Trichome initiation is controlled by the trimeric complex MBW (MYB-bHLH-WD repeat protein) [11,12]. Three genes encoding the R2R3-MYB, bHLH and WD repeat protein transcription factors, respectively, play positive regulatory roles in the development of trichome. The corresponding mutant plants (*gl1*, *ttg1* and *gl3/egl3*) all show glabrous and smooth phenotypes [13,14,15]. There are also numerous negative regulators of trichome development, such as CAPRICE (CPC), Triptychon (TRY), ENHANCER of TRY and CPCl (ETC1\ETC2) [16]. Mutation in these genes could increase the density and cluster frequency of trichome in Arabidopsis, indicating that these regulatory factors inhibit trichome development [17]. In addition, the biosynthesis and signal transduction pathway of phytohormone (gibberellin, cytokinin, salicylic acid and jasmonic acid) are involved in the initiation of trichome development [18,19,20]. Compared with Arabidopsis, the regulation mechanisms of multicellular trichome development in other plants still remain unclear; only a few genes have been reported, such as *hair* and *woolly* in tomato [2,21].

The surfaces of all organs in the cucumber generally have trichomes. The trichomes on cucumber ovary/fruits are called spines, which are one of the most important fruit quality traits [22]. Unlike Arabidopsis, trichomes in cucumber are multicellular, usually consisting of three different cell types (head, stalk, and base cells) [23]. In recent years, several genes regulating trichome development in cucumber have been cloned using trichome mutants. Three glabrous mutants of cucumber, *csgl1*, *tbh*, and *mict*, were all caused by mutations in the *CsaV3_3G036660* gene [24,25,26]. The mutation of *CsaV3_3G036660* could also lead to an *nps* (no pyramid shaped) mutant, which exhibited smaller, denser and non-pyramid-shaped head trichomes [27]. The mutation of the HD-ZIP IV transcription factor gene *Tril* (*CsaV3_6G049180*) could induce the occurrence of two other glabrous mutants (*csgl3*, *tril*) in cucumber [23,28,29]. Transgenic expression of the *Tril* gene could partly rescue the mutant phenotypes of *tril*, *csgl3* and *fs1* (*few spines 1*) [30]. The transformation of the *TTG1* gene (*Csa4Gm097650*, WD-Repeat protein), the *GA20ox1* gene (*CsaV3_5G005560*, gibberellin oxidase), the *CsMYB6* gene (*Csa009688*, MYB transcription factor) and the *CsTRY* (*Csa015371*) gene in cucumber showed that all these genes could affect the development of trichomes [25,31,32]. In addition, several genes only related to fruit spines were cloned via map-based cloning technology, such as the *NUMERSOUS SPINES* (*Csa2M264590*, *NS*) gene and the *tender spines* (*CsaV3_1G010120*, *ts*) gene [33,34]. Studies on the genetic relationships revealed a regulatory network that regulates trichome development—*CsTRY* is directly regulated by *CsMYB6* [32]. *CsTTG1* could directly interact with *Mict* [31]. *Mict* is recessive epistasis to the tuberculate fruit gene *Tu* [35]. *Tril* could act upstream of the negative regulator *NS* [30].

Homeobox (HB) genes are highly conserved in plants, and are directly related to homeotic phenomena. The proteins encoded by Homeobox (HB) genes contain a highly conserved Homeodomain (HD) composed of 60 (or 61) amino acids [36]. According to the sequence similarity of homeodomains and the presence of characteristic codomains, HB gene families can be divided into seven types: Knox, DDT, HD-Zip, PHD-finger, ZF-HD, HD-GL2, WOX and HD-BEL type [37]. HB transcription factors positively regulate plant morphogenesis, embryonic development, leaf development, floral organ evolution, plant hormone response, environmental responses to stress, and intercellular transport [38]. The expression of *Knox I* may inhibit the accumulation of auxin and affect leaf formation [39]. The lack of an HB gene (*stm1*) in Arabidopsis leads to the absence of shoot apical meristem in plants [40]. The over-expression of the OsHOX1 transcription factor in Arabidopsis results in short and small leaf phenotypes [41]. It has also been found that Arabidopsis, tobacco and petunia, plants transformed by the HB gene, exhibit phenotypes such as wrinkled leaves, twisted and asymmetric leaves, and ectopic meristems formation [42,43].

In this study, we identified a *multiple trichomes* (*mt*) mutant from an EMS-mutagenized CCMC (North China type) cucumber population. Thus, we detected the candidate gene *CsHD1,* which has never been reported before. Our findings will provide new sight into the molecular mechanisms of cucumber trichome development.

## 2. Materials and Methods

### 2.1. Plant Materials and Mapping Populations

The *multiple trichomes* (*mt*) mutant was obtained from the M_2_ generation of ethyl methanesulfonate (EMS)-treated CCMC plants (Changchunmici, North China type, WT). Wild-type, intermediate-type and *mt* mutant-type plants of the F_2_ generation from the cross between the *mt* mutant and WT plants were used for phenotypic data collection, genetic analysis and BSA-seq. A F_2_ (*n* = 119) segregated population from the cross between the *mt* mutant and “hazerd” (European greenhouse inbred line with normal trichomes) was used to carry out the genetic analysis and primary mapping of the *mt* gene. Then, we expanded the population to 918 individuals for fine mapping. All materials were grown in a greenhouse at Baima Cucumber Research Station of Nanjing Agricultural University, Nanjing, China.

### 2.2. Cryo-Scanning Electron Microscopy

The fresh young leaves of WT, F_1_ and *mt* mutants were frozen in liquid nitrogen for 30 s. The samples were then observed using a Hitachi SU8010 field emission scanning electron microscope.

### 2.3. BSA-Seq Analysis of mt Mutant

Genomic DNA was extracted from young leaves of F_2_ individuals from the cross between *mt* and WT. For whole genome re-sequencing, the equal amount of DNA from 29 WT and 27 *mt* mutant or intermediate types were bulked to generate the wild-type and mutant pool, respectively.

A Truseq Nano DNA HT Sample Preparation Kit was used to generate sequencing libraries (Illumina, Ipswich, Massachusetts, MA, USA). The constructed libraries were sequenced by the Illumina HiSeq4000 platform and 150 bp paired-end reads were generated with insert sizes of around 350 bp. Short reads obtained from the wild type parent “CCMC”, which was re-sequenced in our previous work [44], were aligned against the cucumber genome sequence (‘Chines Long” v3 reference genome) [45] to obtain the consensus reference sequence using BWA software. Reads from the mutant and WT pools were separately aligned to the “CCMC” consensus reference sequence reads to call SNPs (single-nucleotide polymorphisms) using the SAM tools software. Aligned data were passed through a filter to reduce spurious SNP calls caused by sequencing and alignment errors. SNP indices and the Δ (SNP index) were calculated to determine the causal SNPs. The average SNP index and P-value in Chi-square tests for the SNPs in a certain genomic interval were calculated using a sliding window analysis with 2 Mb window size and 10 kb increments. 

### 2.4. Genetic Mapping of mt Candidate Gene

Two new F_2_ populations (*n* = 119 and 918) were applied to finely map the *mt* locus from a cross between *mt* and “hazerd”, which was re-sequenced in our previous work [45]. Indel (insertion deletion) and SNP markers were developed within the initial interval using the re-sequencing data from the parents “hazerd” and “CCMC”. Linkage analysis of the *mt* locus with molecular markers was performed by JoinMap 4.0. Candidate SNPs in the final candidate interval were annotated using the “Chines Long” v3 reference genome via annovar software. All primer sequences are listed in Table S1.

### 2.5. Sequencing and Annotation of the mt Candidate Gene

Total DNA was extracted from the leaves of *mt* mutant and WT plants using a Plant DNA Extraction kit (PD, Tsingke Biotech, Beijing, China). The full-length sequences of candidate genes from the *mt* mutant and WT were sequenced by Tsingke Biotech (Tsingke Biotech, Beijing, China). DNAMAN software was used to compare the full-length DNA sequences and the corresponding protein sequences. The DNA sequences of *CsaV3_6G050410* were sequencing among an additional 72 cucumber inbred lines to verify the reliability of the target mutant sequences. Functional annotations of candidate genes were acquired from the Cucumber Genome Database (http://cucurbitgenomics.org/ accessed on 1 February 2021) and NCBI (National Center for Biotechnology Information) databases (https://www.ncbi.nlm.nih.gov/ accessed on 1 February 2021). The functional domains of the protein were predicted by Pfam 31.0 online software (http://pfam.xfam.org/ accessed on 1 February 2021).

### 2.6. Quantitative Real-Time PCR (qRT-PCR) Analysis 

Quantitative real-time PCR (qRT-PCR) was performed on tissue samples from the root, stem, leaf, male flower, ovary, tendril and fruit peels of *mt* mutant plants, WT plants, and their F_1_ progeny at the same growth stage. Total RNA was extracted from leaves of the *mt* mutant and WT plants using Trizol. cDNA was synthesized using a Prime Script TM RT Reagent Kit (TaKaRa) following the manufacturer’s instructions. Quantitative real-time PCR was performed with an SYBR Premix Ex TaqTM Kit (TaKaRa) in a Bio-Rad CFX96 quantitative real-time PCR system, and the values from triplicate reactions were averaged. The threshold cycle (Ct) value of each gene was investigated and normalized to the Ct value of *CsActin*. To determine relative expression fold differences for each gene during different treatments, the 2^−ΔΔCt^ formula was applied. The primer sequences are listed in Table S1.

### 2.7. Sequence Alignments and Phylogenetic Tree Construction 

HB homologous protein sequences from diverse plant species were obtained using a BLASTP search in NCBI (https://www.ncbi.nlm.nih.gov/ accessed on 1 March 2021). Multiple alignments of protein sequences were performed using DNAMAN software. A neighbor-joining tree was constructed with the MEGA7.0.21 software using the neighbor-joining method based on 1000 bootstrap replications [46]. 

### 2.8. Digital Gene Expression Analysis

Young leaves of WT and *mt* mutant were collected for RNA sequencing analysis with three biological replicates for each genotype. The RNA-Seq libraries were loaded on an Illumina HiSeq 4000 platform and a 150 bp pair-end read was produced. After removing low-quality reads and those containing adapter poly-N, the paired-end clean data were aligned to the cucumber “Chinese Long” v3 reference genome to get the read counts value by HISAT2 software. The FPKM value of each gene in each sample was calculated by the feature Counts software. The R package DESeq2 was used to identify differentially expressed genes (DEGs) between the WT and the *mt* mutant according to a threshold criterion for significantly different expression: Padj < 0.05 and |log2foldchange| > 1. The GO term and KEGG enrichment analysis were conducted for DEGs. 

## 3. Results

### 3.1. Morphological Characterization and Inheritance of a Multiple-Trichomes Mutant in Cucumber

**Figure 1:**
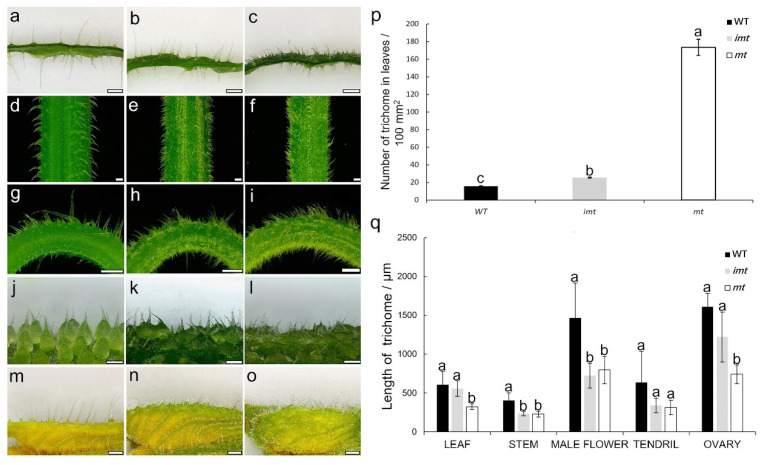
Trichome phenotypic characterization of WT, *mt* mutant and their F_1_ progeny. (**a**,**d**,**g**,**j**,**m**) CCMC (WT). (**b**,**e**,**h**,**k**,**n**) F_1_ progeny crossed between CCMC and *mt*. (**c**,**f**,**i**,**l**,**o**) the *mt* mutant. Bar = 500 μm in (**a**–**i**); bar = 750 μm in (**j**–**l**); bar = 1 mm in (**m**–**o**). (**p**) The number of trichomes on leaves of the mutant, WT and their F_1_. (**q**) The length of trichomes of the mutant, WT and their F_1_. Letters above the bars indicate significant differences at *p* < 0.05.

**Figure 2:**
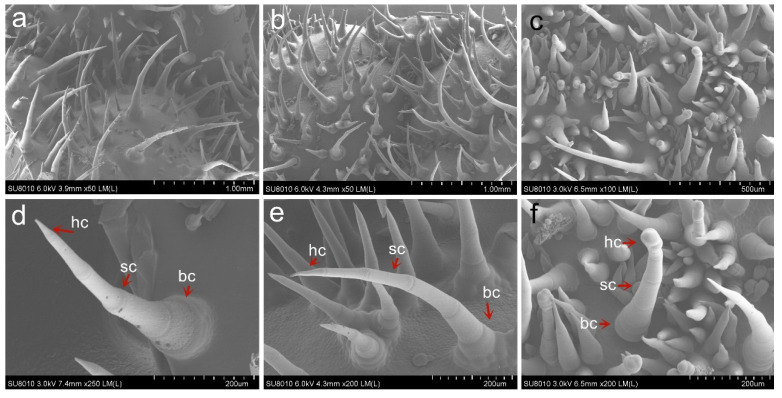
Cryo-SEM images of the leaf of WT, *mt* mutant and their F_1_ progeny. Young leaves on the WT (**a**,**d**), F_1_ progeny (**b**,**e**) and *mt* mutant (**c**,**f**) were observed. Hc = head cell, sc = stalk cell, bc = base cell.

Compared with the WT cucumber, the *mt* mutant cucumber had a higher density of trichomes covering the surfaces of its leaf, stem, tendril, flower organ and ovary (Figure 1). The number of trichomes on the *mt* mutant leaves was about 10.9 times that on the WT. There were approximately 15.6 trichomes per 100 mm^2^ on the leaf of the WT, while there were 173.6 per 100 mm^2^ trichomes on the leaf of the *mt* (Figure 1p). The F_1_ plants, from the cross of WT and *mt*, exhibited intermediate phenotypes and contained a medium amount of trichomes on their surface (25.6 trichomes per 100 mm^2^). The length of trichome of the *mt* mutant was shorter than that of the WT, especially in the ovary, where the average length of trichomes of the *mt* was 742.5 μm and that of the wild type was 1609.9 μm (Figure 1q). The length of the trichomes of F_1_ was intermediate between those of the two parents. In addition, the *mt* mutant showed abnormal plant development, with dwarf, wrinkled leaves and smaller organ phenotypes (Figure S1). Scanning electron microscopy revealed that the number, size, arrangement and shape of trichomes on the *mt* leaf were significantly different from those in the wild type. In addition to the increase in density and decrease in length of trichomes, some of the base cells of the trichome were connected, and the head cells were changed from sharp to pie shaped (Figure 2).

To determine the inheritance pattern of the *multiple trichomes* trait, two F_2_ populations were constructed from the cross of the WT and *mt* mutant, and the hazerd and *mt* mutant. The ratios of plant types of *mt* mutant:intermediate:WT in two populations were 28:88:30 and 20:75:24, respectively, which are consistent with the ratio of 1:2:1 in the Mendelian inheritance law (*p* = 0.0446, 0.0154). This finding suggests that the *multiple trichomes* trait is controlled by an incomplete dominance gene.

### 3.2. Linkage Analysis Identified a Candidate Mutation Site for mt Mutant

**Figure 3:**
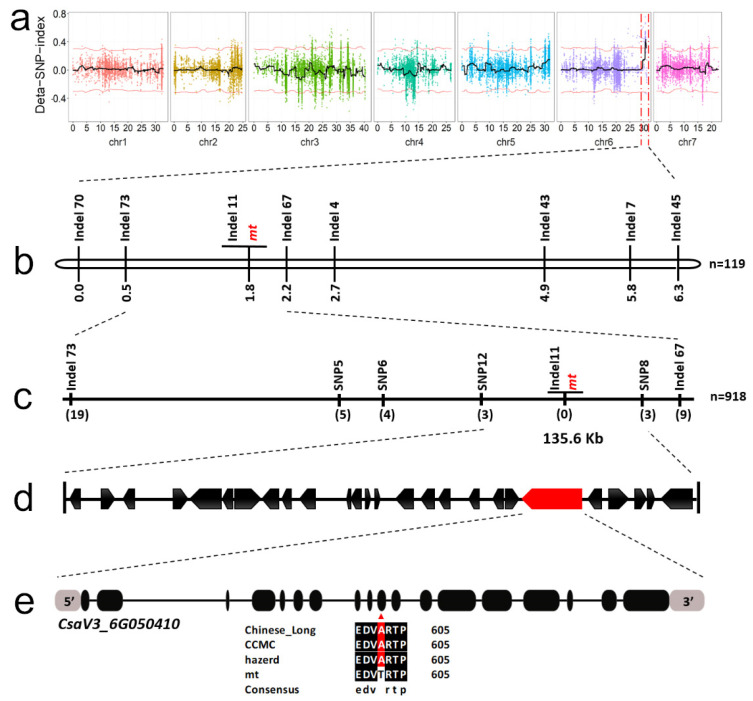
Linkage mapping of the *mt* locus. (**a**) BSA-seq mapped the *mt* locus to one end of chromosome 6. The black curve represents the ∆ (SNP-index), and the red curve represents the 95% threshold line. (**b**) A genetic map that delimits the *mt* locus to a 1.8 cM region. (**c**) High-resolution map for the *mt* locus in a 135.6 kb region. (**d**) Physical position of the mapping region. The black arrows indicate genes in the interval. (**e**) The annotated candidate gene *CsaV3_6G050410* within the *mt* locus. Black rectangles and black lines represent the exon–intron structure, respectively. An SNP in the tenth exon (red arrow) of *CsaV3_6G050410* resulted in an amino acid change from A in Chinese long, CCMC and hazerd to T in *mt* mutant.

BSA-seq was applied to identify the causal gene of the *mt* mutant. Two DNA bulks, the WT and the mutant pools were separately sequenced to generate paired-end reads. 119,338,6482 and 112,581,959 clean reads were mapped to the reference genome in the WT and mutant bulks, respectively. The sequencing depths of the two pools were 54.54- and 51.84-fold, respectively (Table S2). After calculating the SNP index of the two bulks, the Δ (SNP index) was obtained by subtracting the SNP index of WT bulk from the SNP index of mutant bulk. The Δ (SNP index) analysis placed the *mt* locus within a ~1.3 Mb interval in chromosome 6, with a peak of the SNP-index greater than the threshold line (Figure 3a; Figure S2). 

A new F_2_ population (*n* = 119) was constructed from a cross between the *mt* mutant and “hazerd” to narrow down the candidate region. Polymorphic markers in the initial interval were developed by screening the re-sequencing data of WT and hazerd. Seven polymorphic Indel (insertion–deletion) markers were applied to genotype the 119 F_2_ individuals. An initial linkage map was constructed and the *mt* locus was localized within the 1.7 cM region flanked by Indel67 and Indel73 (Figure 3b). A large F_2_ population comprising 918 plants was adopted to further map the target gene. Indel67 and Indel73 were applied to identify 28 recombinants from 918 F_2_ individuals. Four polymorphic SNP markers were developed and used for genotyping the recombinants. Consequently, the *mt* locus was delimited within a 135.6 kb region on chromosome 6 (29,285,654–29,421,297 bp) (Figure 3c).

According to the “Chinese Long v3” cucumber genome database, there were 26 predicted genes annotated in the candidate region (Figure 3d). Based on the re-sequencing data of mutant bulk, WT bulk and CCMC, there were six SNPs detected in this region (Table S3). Among them, four mutation sites that were located in the intergenic region or that caused synonymous variants were filtered out. For the remaining two SNPs, the index of one SNP in the mutant pool was 1, which did not accord with the characteristics of the candidate sites (should be close to 0.67). Only one SNP (*SNP-29389575*) was in accord with these criteria, which caused a nonsynonymous mutation between *mt* and WT, indicating that this SNP was the causative site for the *mt* mutant.

### 3.3. A Mutation in the HD-DDT Gene Resulted in the mt Mutant

The *SNP-29389575* was located in the tenth exon of gene *CsaV3_6G050410*, which is the most likely candidate for the *mt* locus (Figure 3e). The full-length DNA of *CsaV3_6G050410* was sequenced from the *mt* mutant and WT. The *CsaV3_6G050410* DNA was 11352 bp in samples isolated from both the *mt* mutant and the WT. *CsaV3_6G050410* contained 18 exons and 17 introns. Sequence alignments revealed one “G” to “A” base substitution at position 1803 bp in exon 10 of *CsaV3_6G050410*, resulting in an amino acid substitution from A (Alanine) to T (Threonine), corresponding to *SNP-29389575* (Figure 3e). Besides this, the single nucleotide mutation of the candidate gene was only present in the *mt* mutant, and not in the other 72 cucumber inbred lines (Figure S3)

**Figure 4:**
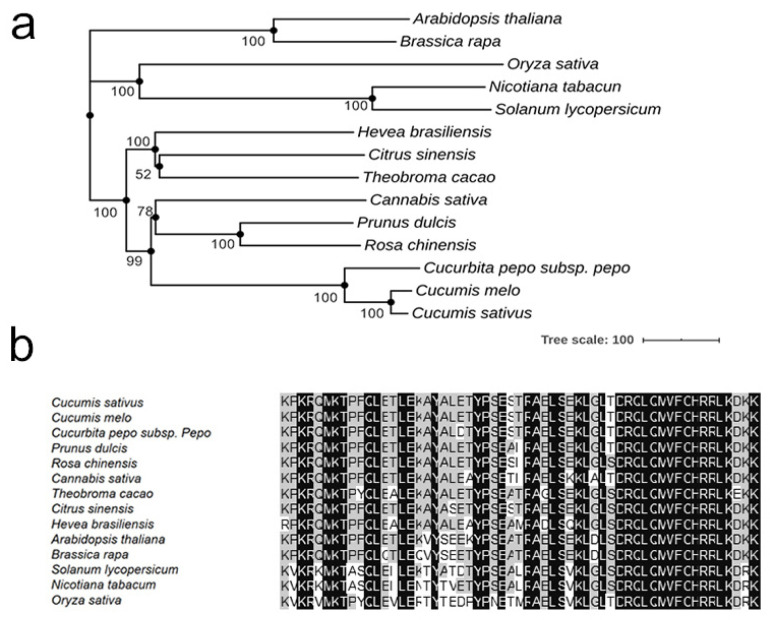
Phylogenetic relationships of cucumber CsHD1 and CsHD1 homologs with other selected plant species. (**a**) A neighbor-joining tree for HD-DDT transcription factor sequences with MEGA 7.0. The numbers at the branch points represent bootstrap values (%) of 1000 replications. (**b**) The conserved homeobox domain of DHD homologs.

The deduced amino acid sequence of *CsaV3_6G050410* contained 1749 amino acids, and was predicted to encode an HD-DDT transcriptional regulator protein containing a conserved homeobox domain (from 32 to 86 aa), a DDT domain (DNA binding homeobox and different transcription factor domain, from 542 to 597 aa), a HARE-HTH domain (from 723 to 791 aa), a WHIM1 domain (from 930 to 974 aa) and a WSD domain (from 1101 to 1172 aa). The gene was named *CsHD1*. Then, 14 *HD1* protein sequences from cucumber, melon, Arabidopsis, tomato, tobacco, rice, rose and other plants were used to construct a neighbor-joining (NJ) tree. The results reveal that *CsHD1* showed a closer relationship to melon and zucchini HD proteins. The alignment of 14 homologous protein sequences revealed a high degree of conservation of the homeobox domain (Figure 4b).

### 3.4. Expression Pattern of CsHD1 in Cucumber

**Figure 5:**
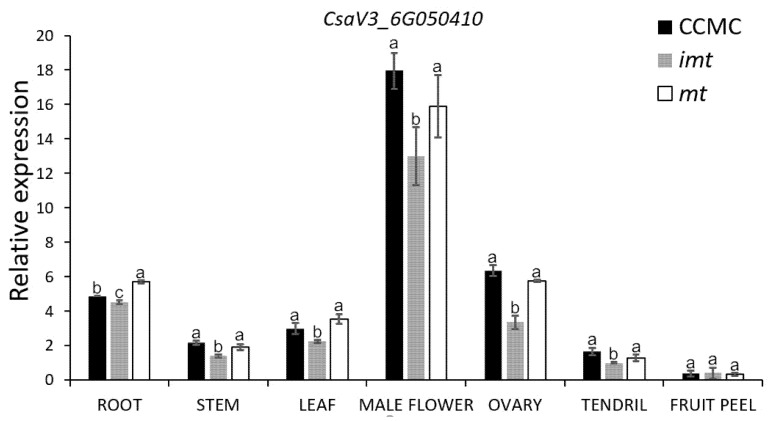
Relative expression level of *CsaV3_6G050410*. Relative expression of the *CsaV3_6G050410* gene in the root, stem, leaf, male flower, ovary, tendril and fruit peel of the WT, the *mt* and their F_1_ progeny (*imt*) by qRT-PCR. Data are displayed using the Actin gene as an internal control with three biological and three technical replicates. Values are the mean ± SD. Letters above the bars indicate significant differences at *p* < 0.05.

The expression level of *CsHD1* was investigated in the root, stem, leaf, male flower, ovary, tendril and fruit peel of the *mt* mutant, the WT and the F_1_ progeny (intermediate *multiple trichomes*, *imt*). *CsHD1* was expressed in all tissues of the *mt* mutant, WT and *imt*, but it was highly expressed in male flowers and lowly expressed in fruit peel (Figure 5). The expression of *CsHD1* in *imt* was lower than in the WT and the *mt* mutant. However, there was no expression difference between *mt* and WT except in the root, in which the expression of this gene was slightly up-regulated in the *mt* mutant (Figure 5).

### 3.5. Transcriptome Profiling Reveals the Key Regulatory Molecules Involved in CsHD1-Dependent Trichome Development in Cucumber

**Figure 6:**
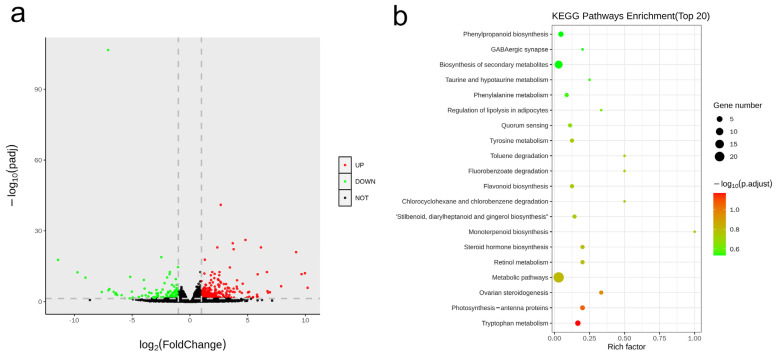
Transcriptome analysis between WT and *mt* mutant. (**a**) Volcano map of differentially expressed genes (DEGs). Red dots indicate the up-regulated genes, green dots indicate the down-regulated genes, and black dots indicate no differentially expressed genes. (**b**) KEGG enrichment analysis of DEGs. The *X*-axis is the enrichment factor score, and the *Y* axis is the enriched pathways. The dot size indicates the number of genes, and the color represents the value of Padj.

To explore the possible mechanism of *multiple trichomes* formation in the *mt* mutant, transcriptome profiling via RNA-seq was performed using the first expanded true leaf of the WT and *mt* mutants. The samples yielded 69.41 to 97.28 million clean reads, which were then mapped to the Chinese Long cucumber v3 reference genome (Table S4). PCA (principal components analysis) revealed significant differences between the two comparisons (Figure S4). Using Padj < 0.05 and |log2foldchange| > 1 as threshold criteria, a total of 486 differentially expressed genes (DEGs) was identified between WT and *mt*, of which 350 and 136 were up-regulated and down-regulated in the *mt* leaf, respectively (Figure 6a). KEGG (Kyoto Encyclopedia of Genes and Genomes) enrichment analysis indicated that these genes were mainly enriched in metabolic pathways, the biosynthesis of secondary metabolites, phenylpropanoid biosynthesis, monoterpenoid biosynthesis, stilbenoid diarylheptanoid and gingerol biosynthesis, steroid hormone biosynthesis, steroid biosynthesis, steroid hormone biosynthesis and monoterpenoid biosynthesis pathways (Figure 6b).

Notably, several metabolite synthesis pathways were enriched, including metabolic pathways, secondary metabolite biosynthesis, and phenylpropanoid biosynthesis. The annotation of DEGs in these pathways revealed that they could encode the Chlorophyll a–b-binding protein, the cytochrome P450 enzyme, flavin-containing monooxygenase, isocitrate lyase, guanylate kinase, caffeoyl-CoA O-methyltransferase, and other proteins (Figure S5). In addition, the majority of the genes were up-regulated in the *mt* mutant.

Because the *CsHD1* gene is a member of the homeobox gene family, the expression of 78 homeobox transcription factor gene family members was explored. Among 78 HB genes, 3 (*CsaV3_1G013780*, *CsaV3_3G002540*, *CsaV3_3G026410*) were found to be up-regulated in the *mt* mutant (Figure S6). Gene annotation showed that they encode the HD-ZIP protein (*CsaV3_3G002540*, *CsaV3_3G026410*) and the BEL1-like HD protein (*CsaV3_1G013780*).

GO (Gene Ontology) term enrichment analysis was also performed, and the results show that DEGs were significantly enriched in “nucleic acid binding transcription factor activity” and “transcription factor activity, protein binding” (Figure S7). In particular, seventy-two transcription factors were identified in the DEGs, including MRKY, MADS, bHLH, MYB and bZIP transcription factors. In addition, the expression of 12 cucumber genes was examined by qRT-PCR to prove the reliability of RNA-seq (Figure S8).

## 4. Discussion

Diverse plant species and tissues of the same species have different physical properties related to plant trichomes [2,3]. Trichomes are found on the epidermis of several organs in cucumbers, and they have a significant impact on the cucumber plant’s growth and development, disease resistance, and fruit yield [22]. Using trichome/spine defect mutants or the transformation of genes associated with trichome development, the regulatory mechanism governing trichome development in cucumber has been reported [24,25,26,27,28,29,30,31,32,33,34]. The related genes are involved in the signal transduction pathways for HD-zip, MYB, and WD40 transcription factors, as well as auxin and gibberellin modulations. Recent studies have revealed the complex regulatory network governing cucumber trichome development [30,31,32,35]. However, further research into the exact mechanism of the linked genes is needed. In comparison to Arabidopsis, cucumber trichome development is barely understood, and more trichome mutant phenotypes need to be investigated. An EMS-induced mutagenesis population yielded a new trichome defect mutant, *mt*, in this investigation. The *mt* mutant had numerous trichomes in various organs compared to the WT. The majority of cucumber trichome defect mutants have a glabrous phenotype, while the *mt* mutant possesses more trichomes (Figure 1). In addition, the trichome growth of the *mt* mutant was more disordered, with shorter trichomes and an uneven distribution (Figure 1).

The cucumber trichome is multicellular and non-glandular, and is composed of three types of cells, including: head cell in a pyramidal shape, stem cell, and base cell [23]. The formation of the cucumber trichome can generally be divided into two steps: the first is the initiation of the development of the stem and base cells followed by expansion of the base cells [24]. There are several kinds of trichome cell in cucumber glabrous mutants *tbh*, *mict*, *csgl1* and *nps*, including those with only one small papillary head cell, those composed of one to five round cells, and those without pyramidal head cells or pie-shaped head cells [24,25,26,27]. Because of differences in cell division and elongation, *tbh*, *mict* and *csgl1* exhibit a glabrous phenotype, while *nps* shows denser trichomes. By cryo-scanning electron microscopy, we found that in the leaf of the *mt* mutant, besides the increase in density, the cell state of the trichome was also changed (Figure 2). The leaf epidermis was densely covered with smaller trichome cells. The head cell of most trichomes changed from a pyramid to a pie shape, but there are also a few kinds of trichomes that can develop into a pyramid shape. 

The *mt* locus was accurately located at the end of chromosome 6 in the cucumber, within a 135.6 kb region, by BSA-seq technology combined with linkage marker analysis (Figure 3). The sequencing depth and coverage of bulked pools could screen the mutation sites in the candidate interval. Through screening, we found that there were six SNP sites between the two bulked pools within this interval (Table S3), and only *SNP-29389575* could meet the screening criteria (the value of SNP index). Gene annotation indicated that *SNP-29389575* was located on exon 10 of gene *CsaV3_6G050410,* and it caused the change in its coding amino acid (Figure 3e). It is worth noting that qRT-PCR analysis showed that the expression level of *CsaV3_6G050410* in the *mt* mutant was similar to that in the WT, indicating that the occurrence of the mutation site *SNP-29389575* did not cause changes in the RNA accumulation of *CsaV3_6G050410* (Figure 5). Therefore, we speculate that *mt* mutation is not caused by transcriptional changes in the *CsHD1* gene, but may be caused by functional changes due to structural differences in its encoded proteins (Figure 3e). In some of the glabrous mutants of cucumber, gene expression was down-regulated and its function was lost, resulting in a series of trichome defect mutants [25,26]. In the *mt* mutant, the change in protein encoded by *CsaV3_6G050410* may enable it to acquire new functions, making the cucumber produce more trichomes.

Two mechanisms mainly regulate the initiation and development of plant trichomes. The initiation of unicellular non-glandular trichomes in Arabidopsis and cotton is controlled by the MYB-bHLH-WD repeat complex [11,12]. Plants with multicellular structure trichomes, such as tomato and maize, have another regulatory mechanism [21,47]. In these plants, the ectopic expression of MYB-bHLH-WD complex-related genes could not change the developmental status of the trichome [21]. The development of trichomes in these plants may be regulated by the plant-specific HD-ZIP protein [48]. Both of these two regulatory mechanisms could play a role in the development of trichomes in cucumber. The over-expression of the MYB transcription factor gene *TRY* or *MYB6* in cucumber could reduce fruit spines. In contrast, the over-expression of the WD-repeat transcription factor gene could increase fruit spines, indicating that the development of cucumber trichome/spine may also be regulated by the MYB-BHLH-WD-repeat complex [32,35]. The loss of function of class I or IV HD-ZIP proteins could result in a smooth glabrous phenotype in the cucumber [23,24]. In this study, the regulatory gene for the *mt* mutant encodes an HD-DTT class transcription factor with multiple domains, including HD and DDT domains. HD transcription factors play different roles, such as regulating embryonic development, responding to the environmental stress of plants, and affecting the evolution of flowers, intercellular material transport and male meiosis [38]. HD-zip transcription factors are essential for plant trichome development [49]. However, there has been no report on the relationship between the HD-DTT transcription factor and plant trichome development. The results of this study confirm that the HD-DTT transcription factor could regulate the number and structure of cucumber trichomes (Figure 4). In addition, transcriptome data revealed that these HB genes were differentially expressed in the mutants, indicating that HD transcription factors play a role in the trichome development of cucumber (Figure S6). 

Transcriptome profiling was used to analyze the possible regulatory pathways of the target gene for the *mt* mutant. There were 486 DEGs in the *mt* mutant, and most of them were up-regulated (Figure 6a). These DEGs were enriched in multiple KEGG pathways. Several metabolite synthesis pathways were enriched, including metabolic pathways, the biosynthesis of secondary metabolites, and phenylpropanoid biosynthesis. Further gene annotation showed that these DEGs were involved in flavonoids synthesis, chlorophyll, terpenes, and other metabolites. The transcriptome analysis of the cucumber trichome mutant *mict* also showed that several genes related to the metabolic pathways of flavonoid and cuticle metabolism were differentially expressed in *mict*, and when further combined with metabolome analysis, this indicates that flavonoids’ and cuticle components’ metabolic pathways are co-regulated by the *mict* gene [50]. The transcriptome analysis of the *mt* mutant indicates that these metabolites (flavonoids, chlorophyll, terpenes, etc.) may play an essential role in trichome development in cucumber.

It is worth noting that a number of hormone-related pathways were enriched, including steroid biosynthesis, steroid hormone biosynthesis and monoterpenoid biosynthesis (Figure 6b). These pathways may affect the synthesis and function of hormones, including gibberellins, brassinolide and cytokinin. Plant hormones are involved in the development of plant trichomes [18,19,20]. For example, ethylene could regulate the microtubule assembly to control the trichome branch, and its external application could increase the branching of the cucumber trichome [51]. Hormone treatments (GA3\Ethylene\IAA\JA) could increase trichome numbers in cucumber fruit [52]. The enrichment analysis of DEGs indicates that the target gene might affect the synthesis or transport of plant hormones to regulate trichome development. In addition, some transcription factors were also differentially expressed, including MRKY, MADS, and bZIP. In Arabidopsis, in addition to the MYB-BHLH-WD-repeat transcription factors, other transcription factor families are also involved in the initiation and development of trichomes [53]. The expression changes of these transcription factor genes in *mt* mutants indicate that they might directly or indirectly regulate trichome development.

In addition to abnormal trichome development, other morphological characteristics of the *mt* mutant were also changed, resulting in dwarfing, leaf shrinkage, fruit shortening and other defective phenotypes, which may be further effects of mutation in the HD transcription factor (Figure S1). The ectopic expression of Knt1 (Knox-like HD transcription factor) in tobacco resulted in drastic phenotypic changes, including abnormally shaped leaves, the loss of apical dominance, and significant dwarfism [43]. The overexpression of the rice *OsHOX1* transcription factor gene in Arabidopsis led to the inhibition of transgenic plant growth, short leaves, and other defective traits [41]. In this study, it was also confirmed that the mutation of the HD transcription factor could affect the growth and development of cucumber plants.

## 5. Conclusions

In this study, we identified a *multiple trichomes* (*mt*) cucumber mutant, with a higher density of trichomes in all organs above ground, including the leaf, stem, tendril, fruit, and flower, compared with the wild-type plant. We described the phenotypic and genetic characteristics of the cucumber *mt* mutant. Fine mapping revealed *CsaV3_6G050410* as the potential candidate gene for *mt*, which encodes an HD-DDT transcriptional regulator protein. These results provide new insights into the genetic mechanism controlling trichome development in cucumber.

## Data Availability

All relevant data analyzed during this study are included in this article and in the additional files. The RNA-seq data used in this study were deposited in the NCBI public database (SRA accession: PRJNA685586).

## Supplementary Materials

The following are available online at https://www.mdpi.com/article/10.3390/genes12101478/s1, Figure S1 Phenotypic images of the *mt* mutant. Figure S2 BSA-seq analysis of *mt* locus. Figure S3 Partial DNA sequence alignment of *CsaV3_6G050410* among 72 cucumber inbred lines, 9930, WT and *mt*. Figure S4 PCA analysis of samples for transcriptome. Figure S5 Expression of genes related to metabolite synthesis in the leaf of *mt* mutants and WT. Figure S6 Expression of HD transcription factor in the leaf of *mt* mutant and WT leaves. Figure S7 GO enrichment analysis of DEGs between the *mt* and WT. Figure S8 Comparison of the expression ratios of select genes by RNA-seq and qRT-PCR. Table S1 Sequences of primers used in the study. Table S2 The whole genome sequencing reports of WT, mutant bulk and WT bulk. Table S3 Annotation of SNPs in candidate interval. Table S4 The whole genome sequencing reports of samples for RNA-seq.

## Abbreviations

CCMC: Changchunmici, North China type; WT: wild type; mt: multiple trichomes; imt: intermediate multiple trichomes; gl1: glabra1; csgl1: glabrous 1; gl3: glabra3; egl3: enhancer of glabra3; ttg1: transparent testa glabra 1; tbh: tiny branched hair; mict: micro-trichome; tril: trichomeless; NS: NUMERSOUS SPINES; ts: tender spines; HB: homeobox; HD: homeodomain; HD-DDT: homeodomain-DDT; Indel: insertion–deletions; SNP: single-nucleotide polymorphism; EMS: Ethylmethylsulfone; BSA-seq: Bulked Sergeant Analysis Sequencing; MYB: myeloblastosis; bHLH: basic helix–loop–helix; MBW: MYB-bHLH-WD-repeat protein; HD-ZIP: homeodomain-leucine zipper; NJ: neighbor-joining; PCA: principal components analysis; DEGs: differentially expressed genes; KEGG: Kyoto Encyclopedia of Genes and Genomes; GO: Gene Ontology; qRT-PCR: quantitative real-time PCR; Ct: threshold cycle; GA3: gibberellin3; IAA: β-indoleacetic acid; JA: jasmonic acid; hc: head cell; sc: stem cell; bc: base cell.
