# Supplementary material for: A SNP Mutation in Homeodomain-DDT (HD-DDT) Transcription Factor Results in Multiple Trichomes (mt) in Cucumber (Cucumis sativus L.)
Source: Genes (Basel). 2021 Sep 23;12(10):1478. doi: 10.3390/genes12101478 (PMC8536133; doi:10.3390/genes12101478)
Supplement: Supplementary file 1 [file genes-12-01478-s001.zip › genes-1281735-supplementary.pdf]

**Supplementary Information**

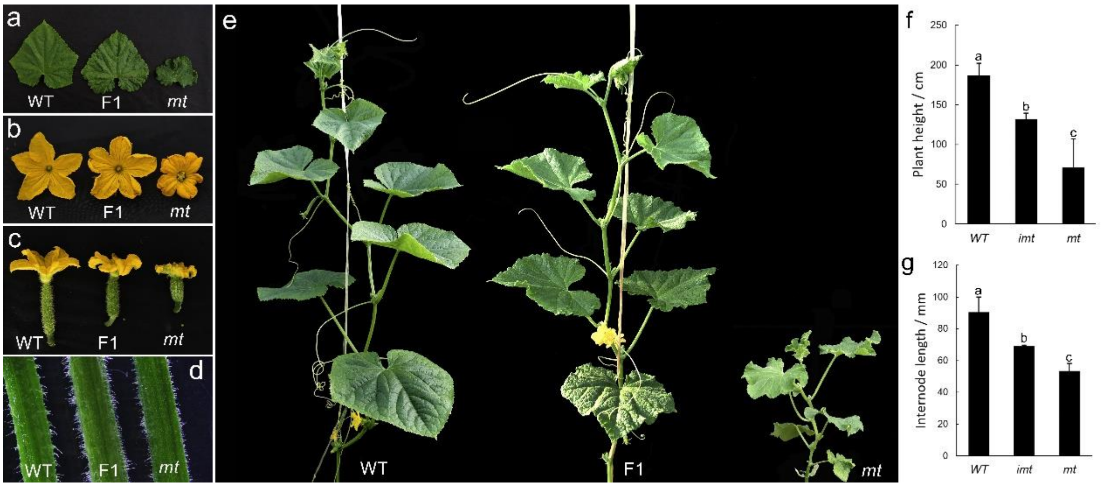


**Fig. S1** Phenotypic images of the *mt* mutant. Representative photographs of *mt* mutant cucumber plants showing different development in leaf (**a**), male flower (**b**), ovary (**c**), stem (**d**), the whole plant at flowering stage (**e**). $F_1$ represents the hybrid generation of *mt* and WT. **f** Plant height of WT, *mt* and $F_1$. **g** Internode length of wild type, mutant and $F_1$. Letters above the bars indicate significant differences at $P < 0.05$.

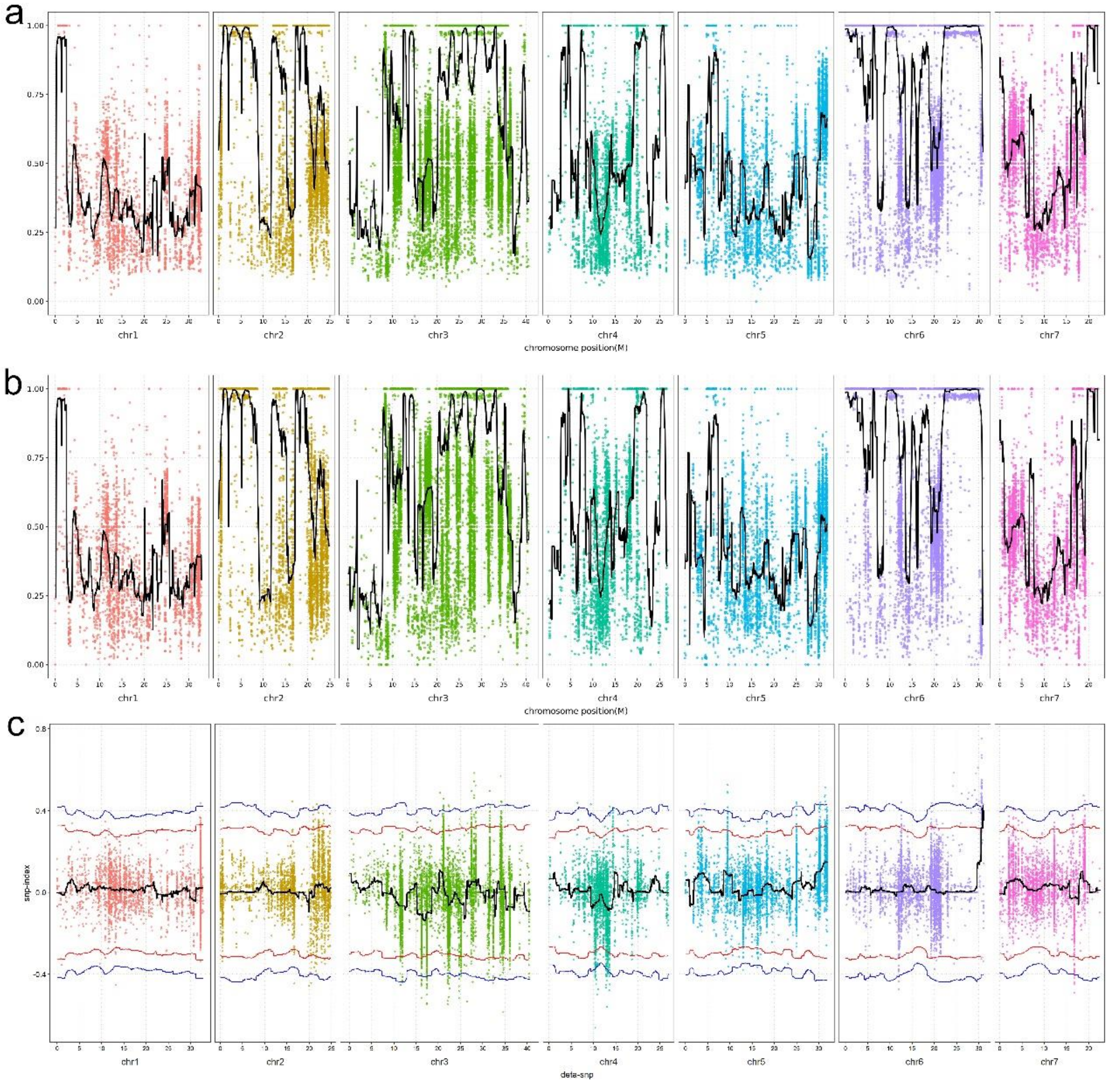


**Fig. S2** BSA-seq analysis of *mt* locus. SNP-index graphs of WT-pool **a**, mutant-pool **b** and Δ (SNP-index) **c** from BSA-seq analysis. The Δ(SNP-index) plot with statistical confidence intervals under the null hypothesis of no SNP (green, P< 0.01; red P< 0.05). The X-axis represents the position of seven chromosomes, and the Y-axis represents the SNP-index.

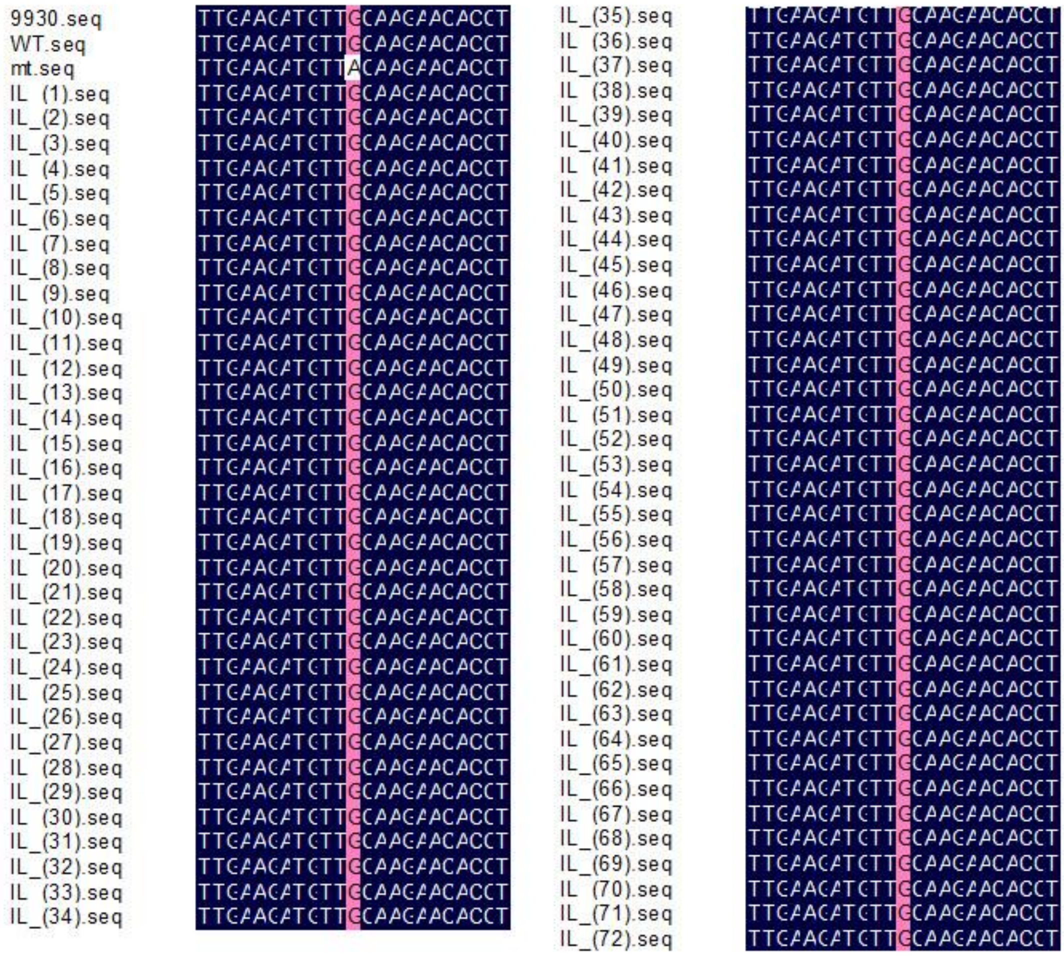


**Fig. S3** Partial DNA sequence alignment of *CsaV3_6G050410* among 72 cucumber inbred lines, 9930, WT and *mt*.

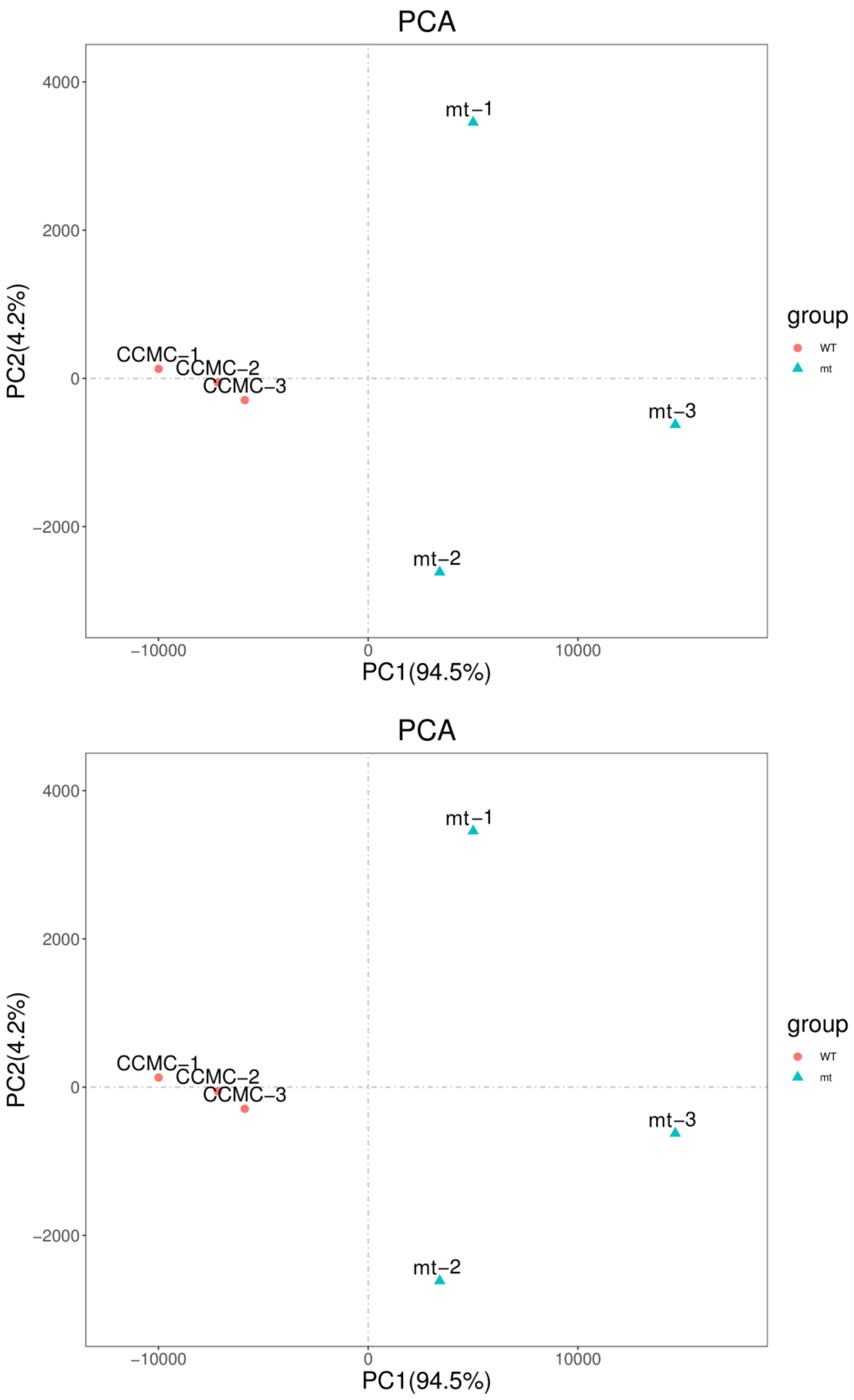


**Fig. S4** PCA analysis of samples for transcriptome. The circle represents the WT sample and the triangle represents the *mt* mutant sample.

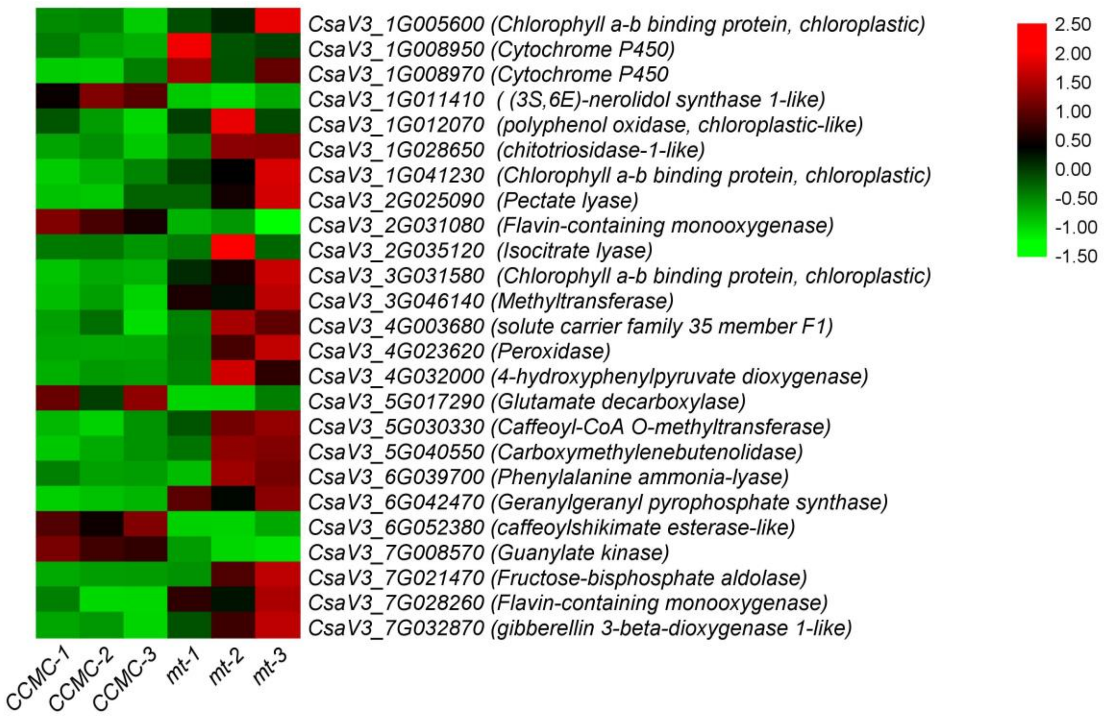


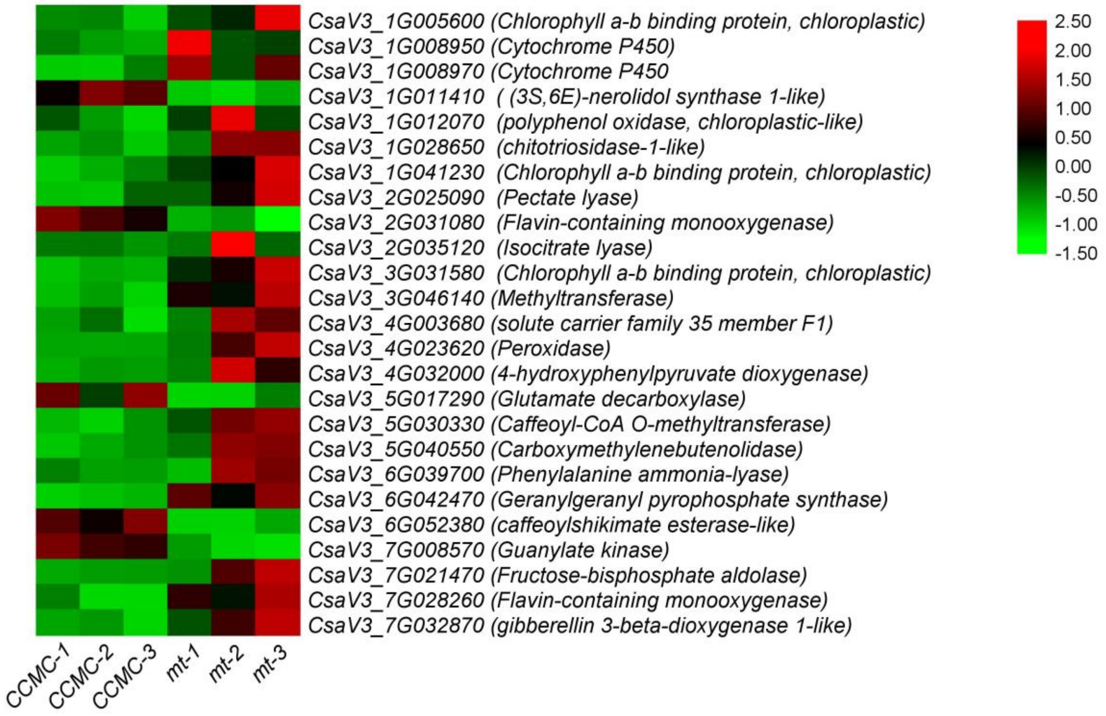


**Fig. S5** Expression of genes related to metabolite synthesis in leaf of *mt* mutant and WT. The expression data were collected from the fkpm value of transcriptome data and standardized by Log2 function.

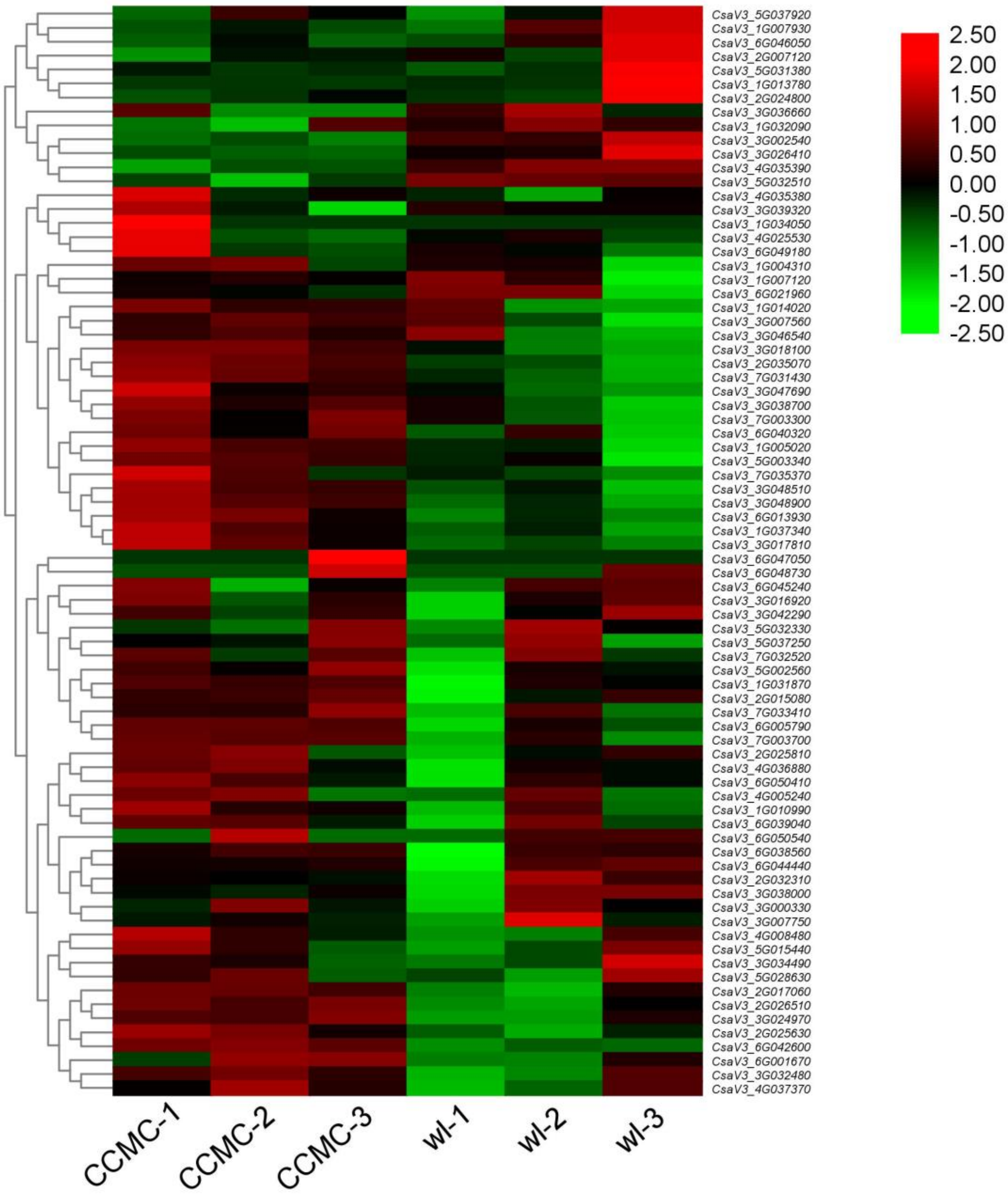


**Fig. S6** Expression of HD transcription factor in leaf of *mt* mutant and WT. The expression data were collected from the fkpm value of transcriptome data and standardized by Log2 function.

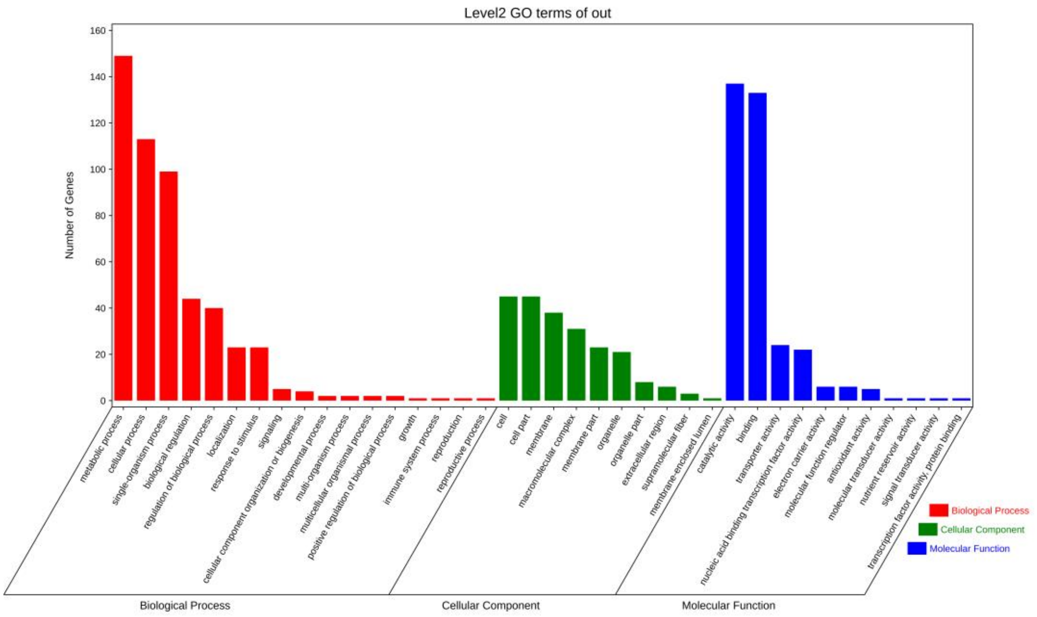


**Fig. S7** GO enrichment analysis of DEGs between the *mt* and WT.

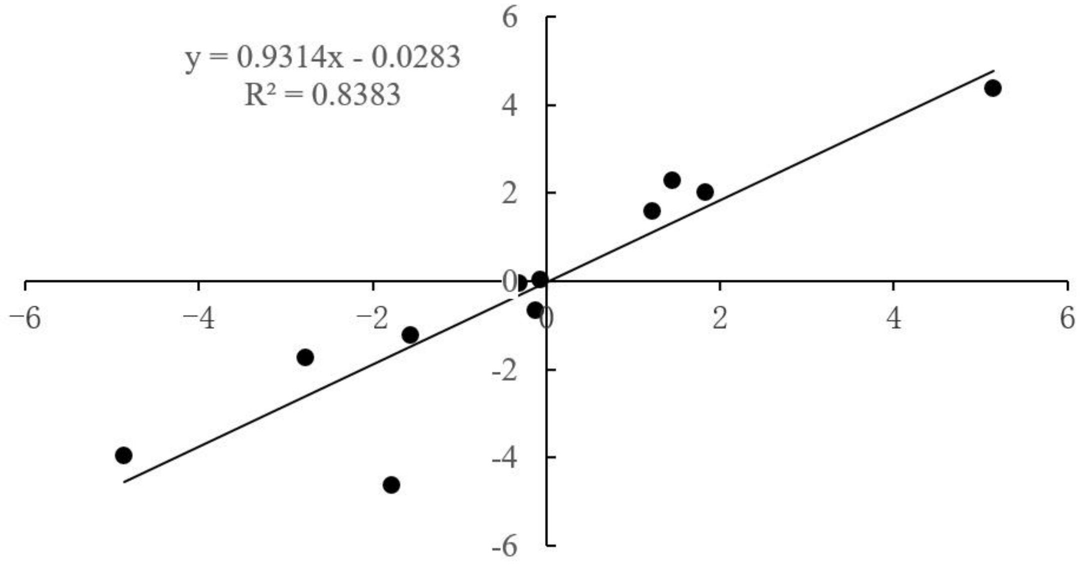


**Fig. S8** Comparison of the expression ratios of select genes by RNA-seq and qRT-PCR.

**Table S1** Sequences of primers used in the study.

| Primer ID | annotation | Forward primer | Reverse Primer |
|---|---|---|---|
| Indel4 | Indel marker | TTAGATTCAAGACCTCAAAC | TGCAGACAGACATATCTCATA |
| Indel7 | Indel marker | TTAGCAATGTCAGGGGCTAC | CAGGGCATGGACTAGTTCAA |
| Indel11 | Indel marker | GTTTTGAATTTGATGGTTCC | GCTGAAAAGTAATTGAGGCT |
| Indel43 | Indel marker | AGCTGGATTGCAATATTTCG | TCCTAGAGGTCTTTGGGGTC |
| Indel45 | Indel marker | GTGTTTGGTTTCAACATTTA | CATATTGTTTTTGCTAGGTA |
| Indel67 | Indel marker | TACTATTGAATTGAGAGAGCG | TGTCAAACGGGTACTGTACT |
| Indel70 | Indel marker | CCACCTATGTCTACACTTTT | ATCAATGAGACCTTTAAAGT |
| Indel73 | Indel marker | TACAGCGACAGAGAAGGATAC | TGGAGGTTATTTACGAAGACT |
| SNP12 | SNP marker | GTCATCTTCGTATAGGGTC | ACACAACTTTACAATCTGG |
| SNP5 | SNP marker | CGTTTGGGATGATTGTTTT | AATGATTTGGTGAGGAGAT |
| SNP6 | SNP marker | ATTCACCTGTCTCACCTACA | CTTAGAAGTCCGAAAATGC |
| SNP8 | SNP marker | TATTTGAAAGGAGAGAGATG | TGATATTTACACTGGCTGTT |
| *CsaV3_6G050410* | Full-length DNA sequencing | TTATCTTCTTCTTCCTCTTCC | CTAAACATACTGCATCGTC |
| *CsaV3_6G050410* | Full-length DNA sequencing | ATTGCCATATTCATGAATCG | GTTTGAACTAGAGAGAAATG |
| *CsaV3_6G050410* | Full-length DNA sequencing | TTTTCCTTAGGATAGGAATC | CCTGTTCAACTGTTCATGAC |
| *CsaV3_6G050410* | Full-length DNA sequencing | GGCTGCAAGAGGATTCCCTG | GACTGACACCAAGTATGACC |
| *CsaV3_6G050410* | Full-length DNA sequencing | ACAGCTTGAACTGATGGAAC | GAGAGACCCATCTTAGAATATAC |
| *CsaV3_6G050410* | Full-length DNA sequencing | TTGCCTTTTGAGACTGATTG | TTCTTCATCTCTTTCAACATC |
| *CsaV3_6G050410* | Full-length DNA sequencing | GAAAGAATAGCTCCTTCCAC | CCTCTTCAGAATCAATAAGC |
| *CsaV3_6G050410* | Full-length DNA sequencing | GCTGAAGAAATGTACACATAC | GTAGAAGCTCCTCAATAGAA |
| *CsaV3_6G050410* | Full-length DNA sequencing | TAGACTTAAAGG | CATCAGATCATCAT |

| | | | |
|---|---|---|---|
| | | CTAATGCC | AATCCAAG |
| *CsaV3_6G050410* | Full-length DNA sequencing | TATTAGAGAATTT ATAACAGC | ATGCTTAAGGGTC TGTAATTG |
| *CsaV3_6G050410* | qRT-PCR | GATAACAGCGCA ATGGAGAATT | CTTCTGAATTAGC AGCTTCACC |
| *Csa6M484600.1* | Actin | TTCTGGTGATGGT GTGAGTC | GGCAGTGGTGGTG AACATG |

**Table S2** The whole genome sequencing reports of WT, mutant bulk and WT bulk.

| Sample | Mapped reads | Total reads | Mapping rate(%) | Average depth(X) | GC Content(%) | Coverage at least 4X(%) |
|---|---|---|---|---|---|---|
| CCMC | 41,218,820 | 48,655,228 | 84.72 | 16.46 | 32.45 | 96.28 |
| WT bulk | 119,338,648 | 131,949,152 | 90.44 | 54.54 | 38.72 | 98.76 |
| Mutant bulk | 112,581,959 | 127,023,078 | 88.63 | 51.84 | 38.09 | 98.75 |

**Table S3** Annotation of SNPs in candidate interval.

| **Chr** | **Pos.** | **Ref** | **Alt** | **m-index** | **w-index** | **Detaindex** | **Annotion** | **Gene** |
|---|---|---|---|---|---|---|---|---|
| chr6 | 29311543 | A | G | 1 | 0.966667 | 0.033333333 | intergenic regions | None |
| chr6 | 29333906 | G | A | 0.973684 | 1 | -0.026315789 | intergenic regions | None |
| chr6 | 29389575 | C | T | 0.631579 | 0.05 | 0.581578947 | missense_variant | *CsaV3_6G050410* |
| chr6 | 29396706 | G | A | 0.977273 | 1 | -0.022727273 | intergenic regions | None |
| chr6 | 29411393 | C | T | 0.942857 | 1 | -0.057142857 | synonymous_variant | *CsaV3_6G050440* |
| chr6 | 29415880 | A | G | 1 | 0.970588 | 0.029411765 | 3_prime_UTR_variant | *CsaV3_6G050450* |

**Table S4** The whole genome sequencing reports of samples for RNA-seq.

| Sample name | Clean_reads | Total mapped | Q20(%) | GC(%) |
|---|---|---|---|---|
| CCMC-1 | 89191552 | 84682936(94.95%) | 96.42 | 43.52 |
| CCMC-2 | 92916126 | 87954260(94.66%) | 96.2 | 43.91 |
| CCMC-3 | 97288334 | 92295987(94.87%) | 96.41 | 43.77 |
| *mt*-1 | 69411724 | 65444471(94.28%) | 96.44 | 43.91 |
| *mt*-2 | 77349816 | 73317518(94.79%) | 96.25 | 44.09 |
| *mt*-3 | 76718978 | 72598593(94.63%) | 96.43 | 44.23 |
